# Epidural steroid injections in lumbar disc herniation- Evidence synthesis from 72 randomised controlled trials (RCTs) and a total of 7701 patients

**DOI:** 10.1016/j.bas.2025.104216

**Published:** 2025-03-13

**Authors:** Alexandros G. Brotis, Theodosios Spiliotopoulos, Adamantios Kalogeras, Kostas N. Fountas, Andreas K. Demetriades

**Affiliations:** a University Hospital of Larissa, Larissa, Thessaly, Greece; b School of Health Sciences, University of Thessaly Volos, Thessaly, Greece; c Department of Neurosurgery, Royal Infirmary Edinburgh, Edinburgh, UK; d Department of Neurosurgery, Leiden University Medical Center, Leiden, The Netherlands

**Keywords:** Epidural steroid injection (ESI), Sciatica, Lumbar disc herniation, Nerve root block, Systematic review, Conservative treatment

## Abstract

**Introduction:**

The role of epidural steroid injection in treating sciatica still remains debatable.

**Research question:**

To compare epidural steroid injection with other manipulations in terms of pain control, quality of life and other parameters (Q1), compare the various available ESI alternatives regarding the approach (Q2), compare ESI to analgesia (Q3), identify the ideal ESI protocol (Q4), compare different guiding techniques (Q5) and determine the role of ESI as e predictive factor for the outcome.

**Material and methods:**

This systematic review searched three databases from inception to February 2024. Independent reviewers assessed and gathered the data and also the quality of evidence was critically appreciated.

**Results:**

The systematic review included 72 randomized controlled trials 7701 patients. There was a big variation among the aim of the studies. ESI proved to surpass other conservative methods for treating sciatica, however it does not provide long-term results. US- guided as well asFL-guided ESI was proved to have superior results. On the other hand, the role of ESIs in predicting the requirement for surgery is understudied. Comparing the different approaches in ESI the TFESI proved mostly to have better results.Surgery still remains the method with the most instant results providing also long-term treatment.

**Discussion and conclusions:**

ESI has superior results to other conservative treatment modalities for sciatica; However surgery seems to have more immediate effect and better long term outcome. Apart from different approaches, additional agents such as amitriptyline proved to have effect when administered additionally to ESI. More studies need to be conducted for ESI as a predictive factor for the outcome or need of surgery.

## Highlights

• Εpidural steroid injections can significantly alleviate pain and improve functional outcomes for patients with sciatica.
• There are different modalities to be applied in order to treat lumbar disc herniation.
• There is ongoing debate on the optimal method and substance in epidural steroid injection.
• Surgery still has superior results than other manipulations.

## Abbreviation list

ACS: autologous conditioned serum
BMI: body mass index
CESI: caudal epidural steroid injections
CT-guided: computed tomography-guided
ESIs: epidural steroid injections
Fl-guided: fluoroscopy-guided
TFESI: transforaminal epidural steroid injections
ILESI: interlaminar epidural steroid injections
NSAIDs: non-steroidal anti-inflammatory drugs
LBP: low-back pain
LDH: lumbar disc herniation
ODI: Oswestry disability index
ODQ: Oswestry Low Back Pain Disability Questionnaire
PELD: percutaneous endoscopic lumbar discectomy
PIL: parasagittal interlaminar
PPD: plasma disc decompression
PRF: pulsed radiofrequency
SLRT: straight leg raising test
RCTs: randomized controlled trials
SF-36: 36-Item Short Form Health Survey
US-guided: ultrasound-guided
VAS: visual analog scale

## 1 Introduction

Sciatica is a common and debilitating symptom of lumbar disc herniation (LDH), often accompanied by numbness and muscle weakness. While it usually resolves on its own over time without any major consequences, it can cause significant discomfort and disability and have a negative impact on quality of life. Chronic cases of sciatica can lead to work absenteeism and psychological distress. Sciatica, along with low back pain, presents a considerable challenge to healthcare providers and can be costly (see Table 3, Table 4, Table 5, Table 6, Table 7).

The usual care for sciatica involves conservative measures such as adequate pain relief, nonsteroidal anti-inflammatory drugs, and lifestyle modifications. These modifications include bed rest, weight management, physical therapy, and exercise. If the previous measures fail to alleviate the symptoms, physicians may recommend epidural steroid injections (ESIs) as an alternative treatment option. ESIs can help to reduce inflammation and pain associated with lumbar disc herniation. However, it is essential to note that an epidural steroid injection does not directly cause regression of a herniated nucleus pulposus. Moreover, there are important variations in ESIs, including the guiding technique, drug used, dosage, and concentration. This has led to considerable controversy regarding the role of ESI in treating patients with sciatica.

There is ongoing debate regarding the effectiveness of ESIs in treating lumbar disc herniation. While some studies have shown improvements in patient outcomes after receiving these injections, others have found no significant difference. None of the available systematic reviews and meta-analyses focused on high-quality studies only.

The main objective of the present systematic review is to compare ESIs to other treatment modalities such as surgery, manipulations, pulsed radiofrequency (PRF), and "care as usual" in terms of pain control, back-related disability, quality of life, and other outcomes (Q1). Our secondary questions compared the available ESI alternatives, such as interlaminar, transforaminal, caudal, midline, and paramedian approaches (Q2). We also aimed to compare ESI to analgesia (Q3), identify the optimal ESI protocol(drug, concentration, and volume) (Q4), compare different guiding techniques such as fluoroscopy (FL)-, ultrasound (US)-, and computed tomography (CT)-guided techniques (Q5), and determine the response to ESI as a predictive factor for sciatica outcome - surgery (Q6).

## 2 Methodology

We performed a systematic literature review using an electronic search in compliance with the PRISMA checklist. The study did not include any human subjects and was registered in Prospero (international database of prospectively registered systematic reviews in health). This research is independent and not based on any prior systematic review or meta-analysis. The approach we utilised to identify studies in our systematic review is transparently reported in the Electronic Supplementary Material.

### 2.1 Search strategy - information sources

Our current meta-analysis focused on two main concepts: "epidural steroid injections" and "sciatica". Initially, TS and AB searched Medical Subjects Headings/National Library of Medicine, Google Scholar, and Wikipedia to identify relevant keywords. Subsequently, both TS and AB conducted independent searches of electronic databases (PubMed, Scopus, and the Web of Science) using every possible combination of keywords. The search strings for each database are presented in Table 1. Additionally, we searched the reference lists of the collected articles to find additional relevant studies. We conducted a comprehensive literature search on January 6, 2024, and then repeated the search on February 22, 2024 (see Table 2).

**Table 1:** Search strings of our literature review in PubMed, WoS and Scopus.

Database	Search string
PubMed	(“lumbar disc herniation” OR “LDH” OR “lumbar disc herniation” OR “intervertebral disc displacements” OR “disk protrusion” OR “protruded disk” OR “disk prolapse” OR “sciatica” OR “lumbar radiculopathy” OR “lumbar radicular pain”) AND (“epidural steroid injection∗” OR “ESI” OR “epidural steroid∗” OR “epidural corticosteroid injection∗” OR “extradural Injection∗”)
WoS	https://www.webofscience.com/wos/woscc/summary/625331f3-89f6-4fe6-b36b-9a805fe0efaa-cee0e8e1/relevance/1
Scopus	((TITLE-ABS-KEY (“lumbar disc herniation”) OR TITLE-ABS-KEY (“LDH”) OR TITLE-ABS-KEY (“lumbar disc herniation”) OR TITLE-ABS-KEY (“intervertebral disc displacements”) OR TITLE-ABS-KEY (“disk protrusion”) OR TITLE-ABS-KEY (“protruded disk”) OR TITLE-ABS-KEY (“disk prolapse”) OR TITLE-ABS-KEY (“sciatica”) OR TITLE-ABS-KEY (“lumbar radiculopathy”) OR TITLE-ABS-KEY (“lumbar radicular pain”))) AND ((TITLE-ABS-KEY (“epidural steroid injection∗”) OR TITLE-ABS-KEY (“ESI”) OR TITLE-ABS-KEY (“epidural steroid∗”) OR TITLE-ABS-KEY (“epidural corticosteroid injection∗”) OR TITLE-ABS-KEY (“Extradural Injection∗”)))

WoS, Web of Science.

**Table 2:** Baseline characteristics of studies used in Q1.

Author (Year)	Patient	N	Intervention	Comparator	Outcome	Time	Summary	Risk of Bias
Karppinen et al. (2001)	Radicular pain	160	ESI	Saline	VAS (L), VAS (B), patient satisfaction, seek leave, medical costs, and surgery	12 months	Methylprednisolone plus bupivacaine has a temporary impact, but after 3–6 months, patients may experience a "rebound" occurrence.	Low
Vad et al. (2002)	Radicular pain	48	TFESI	Saline	NRS, RMDQ, satisfaction rate	16 months	Fluoroscopic-guided transforaminal injections are a valuable approach to treating lumbosacral radiculopathy caused by a herniated nucleus pulposus	Some concerns
Valat et al. (2002)	Radicular pain	85	ESI	Saline	VAS, Schober's test, SLR-test, RMDQ, and Dallas pain questionnaire	1 month	ESIs are not superior to isotonic saline injections in treating lumbar radiculopathy	Some concerns
Butterman et al. (2004)	Radicular pain	100	ESI	Discectomy; Crossover group	VAS (B), VAS (L), ODI,	3 years	ESI were inferior to discectomy in reducing symptoms and disability associated with a large herniation of the lumbar disc, but were reserved as a short-term conservative measure	Low
Bronfort et al. (2004)	Radicular pain	32	ESI	Self education; spinal manipulation	VAS (L), VAS (B), RMDQ, work absenteeism, and depression	52 weeks	A full scale clinical study comparing all three methods is feasible	High
Dashfield et al. (2005)	Radicular pain	60	CESI (lidocaine plus triamcinolone)	Epiduroscopy (lidocaine plus triamcinolone)	VAS, McGill Pain Questionnaire, Hospital Anxiety and Depression scale	6 months	Targeted steroids via epiduroscopy on the affected nerve root causing sciatica doesn't reduce pain intensity, anxiety, and depression compared to non targeted caudal epidural steroid injection	Some concerns
Wilson - McDonald et al. (2005)	Radicular pain	93	ESI plus local anaesthetic	IM local anaesthetic plus steroid	Pain intensity questionnaire, ODI, SLR-test, Surgery	1 month	ESIs do not affect the ultimate need for surgery in patients in patients with lumbar radiculopathy, but it are useful for reducing symptoms in the acute stages	Low
Bonetti et al. (2005)	Radicular pain	306	ESI	Ozon mixture infiltration	Satisfaction	6 months	Oxygen-ozone treatment achieved superior results compared to ESIs in relieving acute and chronic lower back pain and sciatica	Some concerns
Arden et al. (2005)	Radicular pain	90	ESI	Saline	VAS (B), VAS (L), HAD (anxiety), HAD (depression), SF-36, ODI, medication consumption, and work absenteeism	52 weeks	Although epidural corticosteroid injections may provide some temporary relief, they do not appear to offer long-term benefits to patients suffering from sciatica in terms of pain, function, or the need for surgery.	Low
Zambello et al. (2006)	Radicular pain	351	ESI	Ozon mixture infiltration	VAS, and complications	6 months	Ozone therapy is often the preferred treatment for patients who are not responding to conventional medical management due to its simple administration process, few contraindications, and minimal side effects	Some concerns
Sayegh et al. (2009)	Back and Radicular pain	183	ESI	Saline	ODI and SLR-test	12 months	CEI containing local anaesthetic and steroids demonstrated better and faster efficacy when compared to CEI with	Some concerns
Gerszten et al. (2010)	Radicular pain	90	TFESI	Plasma disc decompression	VAS (L), ODI, SF-36	2 years	PDD achieves a better pain control and an improved functional outcome than those with ESIs	Low
Murakibhavi and Khemka (2011)	Radicular pain	100	CESI	Care as usual	VAS, ODI, BDI, and SLR-test	6 months	Caudal epidural steroid injections appear to yield positive results in treating patients with low back pain and sciatica	High
Iversen et al. (2011)	Radicular pain	116	CESI	Saline, sham injections	VAS (L), VAS (B), ODI, FABQ, pain medication, and EQ-5D	6 months	Caudal epidural steroid injections or saline injections were not recommended for chronic lumbar radiculopathy	Low
Cohen et al. (2012)	Radicular pain	84	ESI	Etanercept; saline	NRS (L), NRS (B), ODI, pain medication, and proceed to surgery	6 months	Epidural steroid injections may provide a short-term but modest pain relief in selected patients with lumbosacral radiculopathy	Some concerns
Cervera- Irimia et al. (2013)	Radicular pain	46	ESI	NSAIDS	VAS, ODI, and satisfaction	6 months	There is no obvious superiority of CESI over NSAIDs in treating chronic low back pain	Low
Shin et al. (2013)	Radicular pain	53	ESI	Saline	NRS, ODI, and satisfaction	6 months	Hypertonic saline has proven to be superior to normal saline as a solvent of triamcinolone in relieving radicular pain in the short term	Low
Spijker-Huigesvet al., (2014)	Radicular pain	66	ESI	Care as usual	NRS, RMDQ	52 weeks	ESI were not recommended, since they had a statistically significant but clinically minor effect on back pain, impairment, and disability in acute radiculopathy	Some concerns
Spijker-Huigesvet al., (2014)	Radicular pain	66	ESI	Care as usual	Costs	52 weeks	Policy makers might consider ESI as an extra treatment option for lumbosacral radicular syndrome, since they have a modest but significant impact on pain and disability, are cost-effective, and carry no reported complications or adverse effects	Low
Sinofsky et al. (2014)	Radicular pain	100	ESI	Saline	VAS (B), VAS (L), ODI, LoS, return-to-work	6 months	Patients experiencing concordant pain during ESIs reported higher pain reduction than patients with non-concordant pain	Some concerns
Spijker-Huiges et al. (2015)	Radicular pain	50	ESI	Care as usual	NRS, RMDQ, SF-36, QALY	4 weeks	Decision-makers may contemplate incorporating ESIs into their daily medical practice in managing lumbar radiculopathy as a means of resource conservation	Low
Shin et al. (2015)	Back and Radicular pain	100	ESI	Saline	VAS (L), VAS (B), ODI, LoS, RtW	4 weeks	Administering epidural steroids following a percutaneous endoscopic lumbar discectomy (PELD) can alleviate both back and leg pain in the immediate postoperative period while enhancing functional outcomes.	Some concerns
Lee et al. (2016)	Radicular pain	18	TFESI	PRF	VAS, ODI, and adverse events	3 months	Administering PRF to a DRG could be equally effective as TFESI in reducing radicular pain caused by disc herniation but without the side effects of steroids	Some concerns
Mehta et al. (2017)	Radicular pain	120	TFESI plus bupivacaine	Care as usual	VAS	1 month	Patients who undergo fluoroscopic-guided Transforaminal Epidural Steroid Injections (TFESI) experience improved pain relief, enhanced quality of life, and reduced need for analgesics compared to those managed conservatively	Low
Nandi and Chowdhery (2017)	Radicular pain	93	ESI	Saline	VAS, SLR-test, Schorber's test, RMI, ODI, and self-rated outcome	4 weeks	CESI seem not to provide any additional improvement over placebo in the natural history of sciatica	Some concerns
Singh et al. (2017)	Radicular pain	40	ESI	SNRB	VAS and ODI	12 months	Caudal epidural block is a safe method, which is less technically demanding and with better pain relief and improvement in functional disability than selective nerve root block	Low
Keorochana et al. (2018)	Radicular pain	30	ESI	Saline	Opioid use, VAS (B), VAS (L), ODI, RMDQ, and complications	6 months	Adding epidural steroids (ES) to percutaneous endoscopic lumbar discectomy (PELD) for patients with lumbar disc herniation does not lead to any significant improvement in postoperative pain, morphine requirement, or disability score	Some concerns
Wilby et al. (2021)	Radicular pain	163	ESI	Microdiscectomy	ODI, VAS (B), VAS (L), RMDQ, COMIS, satisfaction, EQ-5D, and costs	18 weeks	Microdiscectomy seems not to be cost-effective compared with transforaminal epidural steroid injection for sciatica secondary to prolapsed intervertebral disc	Some concerns
Hashemi et al. (2022)	Radicular pain	68	ESI	Autologous conditioned serum	VAS and ODI	6 months	Given the absence of reported complications and its comparable effectiveness, ACS can serve as an alternative to corticosteroids in patients with lumbar radiculopathy	Low
Krahulik et al. (2023)	Radicular pain	150	ESI	Ozon mixture infiltration; Diprophos	VAS	12 weeks	Diprophos had the most effective treatment result as compared to Depomedrone and ozone	Some concerns

ESI: Epidural Steroid Injection; CESI: Caudal Epidural Steroid Injection, TFESI: Transforaminal Epidural Steroid Injection; PRF: Pulsed Radiofrequency; SNRB: Selective Nerve Root Block; DRG: Dorsal Root Ganglion; VAS: Visual Analog Scale; NRS: Numeric Rating Scale; RMDQ:Roland-Morris Disability Questionnaire; SLR: Straight Leg Raise test; ODI: Oswestry Disability Index; HAD: Hospital Anxiety and Depression scale: COMIS: EQ-5D: Questionnaire for health quality assessing 5 domains developed by EuroQol Group; RMI: Repetitive Motion Injuries; LoS: Length of Stay; RtW: Return to Work SF-36:36-item Short Form Health Survey; QALY:Quality-adjusted Life Year BDI: Beck Depression Inventory; NSAIDs: Non-Steroidal Anti Inflammatory Drugs; IM: Intramuscular.

**Table 3:** Baseline characteristics of studies used in Q2.

Author (Year)	Patient	N	Intervention	Comparator	Outcome	Time	Summary	Risk of Bias
Thomas et al. (2003)	Radicular pain	31	TFESI	ILESI	VAS, RMDQ. Dallas pain questionnaire, Schober 's test, finger-to-floor test, SLR-test	6 months	The effectiveness of radio-guided transforaminal epidural corticosteroid injections was found to be greater than that of blindly-performed interspinous injections in treating recent discal radiculopathy	Low
Aref et al. (2007)	Radicular pain	60	ILESI	TFESI	VAS	2 weeks	High concentration and low volume corticosteroid treatment via transforaminal epidural approach for symptomatic lumbar disc herniation resulted in better short-term pain relief and lower need for surgical intervention compared to diluted solution treatment	Some concerns
Jeong et al. (2007)	Radicular pain	239	TFESI (ganglionic)	TFESI (preganglionic)	VAS and self reported outcome	6 months	Transforaminal epidural steroid injection (TFESI) for lumbosacral radiculopathy using a preganglionic approach demonstrates greater efficacy compared to TFESI with a ganglionic approach during short-term follow-up	Some concerns
Ackerman et al. (2007)	Radicular pain	90	CESI	ILESI, TFESI	NRS, ODI, Beck depression scale	24 weeks	TFESIs are more effective than CESIs or ILESIs due to optimized steroid placement in the ventral epidural space.	High
Candido et al. (2008)	Radicular pain	60	TFESI	ILESI	VAS	6 months	The PIL approach may be better suited for routine use than the PIL for placing contrast into the anterior epidural space, resulting in reduced fluoroscopy times and improved distribution	Some concerns
Gharibo et al. (2011)	Radicular pain	38	ILESI	TFESI	NRS, depression, ODI, walking tolerance	3 months	TF epidural steroid injections may provide greater initial relief, although subjective, compared to IL	Low
Ghai et al. (2014)	Back and Radicular pain	62	TFESI	ILESI	VAS, MODQ, side effects and complications	3 months	The PIL approach can be considered a suitable alternative to the TF approach for its equivalent effectiveness, probable better safety profile, and technical ease	Some concerns
Kamble et al. (2016)	Back and Radicular pain	90	CESI	TFESI and ILESI	VAS and ODI	6 months	Transforaminal steroid injections resulted in a better improvement in symptoms both in the short-term and long-term when compared to the interlaminar and caudal steroid injections	Some concerns
Maadawy et al. (2018)	Back and Radicular pain	40	ILESI	TFESI	VAS, MODQ, side effects and complications	6 months	The administration of epidural steroids through the infraneural (IN) technique is considered better than the parasagittal (IL) interlaminar technique due to its superior effect on improving physical function without causing any major side effects	Low
Radoš et al. (2018)	Radicular pain	70	ILESI	TFESI	Quality of sleeping, anxiety, and depression	6 months	TFESI led to lower levels of depression and anxiety and better sleep quality in patients with chronic radiculopathy compared to the ILESI due to a greater reduction in chronic pain	Low
Makkar et al. (2019)	Radicular pain	65	LP-TFESI	M-TFESI	VAS, ODI, BMD, and serum oteocalcin	6 months	The effectiveness of the PIL approach is comparable to that of TF and better than that of the MIL approach for reducing pain and disability in patients with unilateral lumbar radiculopathy	Some concerns
Akram et al. (2021)	Radicular pain	338	CESI	ILESI	VAS (L) and ODI	3 months	interlaminar epidural steroid injections were more effective than caudal ESIs	Some concerns
Ali et al. (2021)	Radicular pain	80	ESI in Kambin space	Conventional ESI	NRS and satisfaction	8 weeks	There is no difference in reducing pain score or patient satisfaction level between using the conventional approach or Kambin's approach	Some concerns
Kumar et al. (2022)	Radicular pain	60	Midline ILESI	Parasagittal ILESI	NRS and ODI	6 months	Pain relief and improvement in disability were clinically better with the parasagittal than the middline interlaminar approach	Some concerns
Singh et al. (2022)	Radicular pain	40	ESI in Kambin space	Conventional ESI	VAS, ODI, patient satisfction, adverse events, complications and failure		There is no difference in reducing pain between using the conventional approach or Kambin's approach	High
Kumar et al. (2023)	Back and Radicular pain	60	ILESI	TFESI	VAS and NRS	4 weeks	TFESIs achieve more satisfiactory pain control than ILESI because it delivers the steroid closer to the pain target	Low
Ozturk et al. (2023)	Radicular pain	60	TFESI	CESI	NRS and ODI	3 months	CESI is as effective as TFESI in reducing pain and disability in S1 radiculopathy; however, CESI requires less radiation exposure and shorter fluoroscopy time	Some concerns

ESI: Epidural Steroid Injection; CESI: Caudal Epidural Steroid Injection, TFESI: Transforaminal Epidural Steroid Injection; ILESI: Interlaminar Epidural Steroid Injection; PIL: Parasaggital Interlaminar; MIL: Midline Interlaminar; TF: Transforaminal; VAS: Visual Analog Scale; NRS: Numeric Rating Scale; RMDQ:Roland-Morris Disability Questionnaire; ODI: Oswestry Disability Index; MODQ: Modified Oswestry Disability Questionnaire; BMD: Bone Mineral Density.

**Table 4:** Baseline characteristics of studies used in Q3.

Author (Year)	Patient	N	Intervention	Comparator	Outcome	Time	Summary	Risk of bias
Ng, Chaudhary, and Sell (2005)	Radicular pain	86	ESI	Bupivacaine	VAS (L), walking distance, ODI	3 months	Adding methylprednisolone to bupivacaine provides no additional improvement in pain control and back-related functional outcome	Low
Tafazal et al. (2009)	Radicular pain	150	ESI plus bupivacaine	Bupivacaine	VAS (L) VAS (B), Zung depression scale, low back outcome score, and ODI	12 months	Adding corticosteroids to local anaesthetic injection alone does not provide any additional benefit for managing sciatica (periradicular)	Low
Manchikanti et al. (2011)	Back and Radicular pain	120	ESI plus lidocaine	Lidocaine	NRS, ODI, employment status, and opioid use	12 months	Caudal epidural injections with local anaesthetic ± steroids are effective in treating discogenic chronic low back pain without radiculopathy	Some concerns
Aref et al. (2011)	Radicular pain	60	ESI plus bupivacaine (1 ml)	ESI plus bupivacaine (2 ml); ESI plus bupivacaine (3 ml)	VAS, analgesic intake	2 weeks	High concentration and low volume corticosteroid treatment via transforaminal epidural approach for symptomatic lumbar disc herniation resulted in better short-term pain relief and lower need for surgical intervention compared to diluted solution treatment	Low
Ghai et al. (2015)	Back and Radicular pain	69	ILESI (methylprednizolone plus lidocaine)	ILESI (lidocaine)	NRS, MODQ	12 months	PIL with local anaesthetic plus steroids is more effective in relieving back and leg pain	Some concerns
Okmen et al. (2017)	Radicular pain	98	ESI plus bupivacaine	Bupivacaine	VAS and ODI	12 months	Adding steroids to interlaminar injections with bypivacaine improves pain and back-related functional outcomes in patients with multilevel disc disease (no leg pain?)	Some concerns
Ter Meulen et al. (2023)	Radicular pain	141	ESI plus bupivacaine	Bupivacaine; NSAIDS	Survalyzer, RMDQ, EQ-5D-3L	6 months	TFESI decreased the opioid requirements in patients with discogenic radiculopathy	High

ESI: Epidural Steroid Injection; ILESI: Interlaminar Epidural Steroid Injection; TFESI: Transforaminal Epidural Steroid Injection; PIL: Parasagittal Interlaminar; VAS: Visual Analog Scale; NRS: Numeric Rating Scale; RMDQ:Roland-Morris Disability Questionnaire; ODI: Oswestry Disability Index; EQ-5D-3L: Questionnaire for health quality assessing 5 domains with 3 response levels of severity developed by EuroQol Group; MODQ: Modified Oswestry Disability Questionnaire.

**Table 5:** Baseline characteristics of studies used in Q4.

Author (Year)	Patient	N	Intervention	Comparator	Outcome	Time	Summary	Risk of bias
Pirbudak et al. (2003)	Radicular pain	60	ESI	ESI plus amitriptiline	VAS, SLR-test, and ODI	6 months	The addition of amitriptyline to ESIs seems to have a significant positive impact on the patient's quality of life	Some concerns
Owlia et al. (2007)	Radicular pain	84	ESI (methylprednisolone 80 mg)	ESI (methylprednisolone 40 mg)	VAS and complications	3 months	Low dose (40 mg) of methylprednisolone in ESI treatment has been found to be equally effective as high dose (80 mg), with similar outcomes and fewer side effects	Some concerns
Cocelli et al. (2009)	Radicular pain	70	ESI (betamethasone)	ESI (tramcinolone)	VAS, ODI, and SLR-test	6 months	Triamcinolone achieves better short-term pain control than betamethasone	Some concerns
Khan et al. (2010)	Radicular pain	103	ESI (methylprednisolone)	ESI (tramcinolone)	VAS, SLR-test, complications	16 weeks	Both methylprednisolone and triamcinolone are effective in treating discogenic sciatica	Low
Park et al. (2010)	Radicular pain	106	TFESI (dexamethasone)	TFESI (triamcinolone)	VAS, McGill pain questionnaire, ODI	1 month	Triamcinolone seems to be more effective than dexamethasone in treating lumbar radiculopathy	Some concerns
Datta and Upadhyay (2011)	Radicular pain	163	CESI (methylprednisolone)	CESI (triamcinolone); CESI betamethasone)	VAS, RMDQ, SLR-test, finger-to-floor test	12 weeks	The second ESI with triamcinolone and dexamethasole was more effective than betamethasone	Some concerns
Kennedy et al. (2014)	Radicular pain	78	TFESI (dexamethasone)	TFESI (triamcinolone)	NRS, ODI, medications, and surgical intervention rate	6 months	Dexamethasone and triamcinolone have similar effectiveness in treating lumbar radiculopathy, but dexamethasone requires slightly more injections to achieve the same outcomes	Low
Tauheed et al. (2014)	Radicular pain	180	TFESI	TFESI plus clonidine	VAS, satisfaction, and complications	6 months	Adding clonidine to TFESIs provided a dose-related improvement in pain relief	Some concerns
Makkar et al. (2018)	Radicular pain	60	ILESI 4 ml	ILESI 6 ml and ILESI 8 ml	VAS and MODQ	24 weeks	The effectiveness of interlaminar ESI did not improve with an increase in the volume of the injectate from 4 ml to 8 ml	Low
Ye et al. (2018)	Radicular pain	95	ESI	ESI via targeted catheter	VAS (L), VAS (B), ODI, and JOA	4 weeks	Administering ESIs using a targeted catheter seems to improve pain control and functional outcome after radiculopathy	Some concerns
Sencan et al. (2019)	Radicular pain	64	TFESI plus sedation	TFESI w/o sedation	NRS and satisfaction	3 months	Administering TFESI under sedation improves patient and physician satisfaction	High
Thiengwittayaporn et al. (2021)	Radicular pain	88	CESI at 40 ml/min	CESI at 20 ml/min	VAS, Standing tolerance, walking tolerance, satisfaction, and complications	12 weeks	It is recommended to avoid administering ESIs at a fast rate, as it may result in increased discomfort at the injection site	Some concerns
Napoli et al. (2023)	Radicular pain	351	TFESI	TFESI plus PRF	NRS, ODI, and RMDQ	52 weeks	Combining pulsed radiofrequency with TFESI seems to improve pain relief and disability	Low

ESI: Epidural Steroid Injection; CESI: Caudal Epidural Steroid Injection; ILESI: Interlaminar Epidural Steroid Injection; TFESI: Transaforaminal Epidural Steroid Injection; PRF: Pulsed Radiofrequency; VAS: Visual Analog Scale; NRS: Numeric Rating Scale; RMDQ:Roland-Morris Disability Questionnaire; ODI: Oswestry Disability Index; MODQ: Modified Oswestry Disability Questionnaire; JOA: Japanese Orthopaedic Association score; SLR: Straight Leg Raise test.

**Table 6:** Baseline characteristics of studies used in Q5.

Author (Year)	Patient	N	Intervention	Comparator	Outcome	Time	Summary	Risk of bias
Park et al. (2013)	Radicular pain	123	US-guided CESI	Fluro-guided CESI	NRS, Odi, and complications	12 weeks	Ultrasound-guided ESIs are equally effective to fluoroscopy-guided ESIs but help may avoid complications induced by intravascular drug administration	Some concerns
Darrieutort-Laffite et al. (2015)	Radicular pain	80	US-guided ESI	ESI	VAS and comlications	NR	US of the lumbar spine was feasible in old and obese patients with lumbar conditions adequately visualizing the epidural space but did not seem to reduce periprocedural discomfort	Some concerns
Elashmawy et al. (2020)	Radicular pain	121	US-guided ESI	Fluro-guided ESI	VAS, ODI, SLR-test, mS-test	3 months	US is equally effective to FL in guiding CESI with excellent treatment outcomes	Some concerns

ESI: Epidural Steroid Injection; CESI: Caudal Epidural Steroid Injection; US: Ultrasound; FL: Fluoroscopy; VAS: Visual Analog Scale; NRS: Numeric Rating Scale; ODI: Oswestry Disability Index; SLR: Straight Leg Raise test.

**Table 7:** Baseline characteristics of studies used in Q6.

Author (Year)	Patient	N	Intervention	Comparator	Outcome	Time	Summary	Risk of bias
Radcliff et al. (2012)	Radicular pain	607	ESI	No ESI	ODI, Worker's compensation, surgery	4 years	ESIs offered no improvement in short or long-term outcomes in patients with radiculopathy but seemed to minimize the requirements for surgery	Some concerns

ESI: Epidural Steroid Injection; ODI: Oswestry Disability Index.

### 2.2 Eligibility criteria - study selection

We conducted a search for randomised controlled trials (RCTs) in English that involved adults aged 18 and over suffering from sciatica and treated with ESI. There were no restrictions on the comparator, the registered outcome, or the duration of the study. However, we excluded studies not written in English and those not designed as RCTs. We uploaded the metadata from all three databases to Rayyan (Tool that helps work quickly through systematic literature reviews) so that the relevant studies could be selected. Initially, we removed all duplicate studies in the three databases. In cases where multiple studies were based on the same study population, only the most recent article was considered. Then, we screened the articles based on the relevance of the title and abstract. We excluded studies based on their study design, such as observational studies, laboratory studies, case series and reports, reviews, editorials, letters to the editor, and the population under study (including children, patients with low back pain only, with stenosis). Finally, we also excluded studies that did not contain extractable data. Any disagreements between the two review authors were discussed with the senior authors (KNF and AKD).

### 2.3 Data collection process

The studies were identified by the first author's name and the year of publication. Two review authors (TS and AB) independently collected the data from each eligible study, including study design, sample characteristics, sociodemographic data, intervention and comparison group details, outcomes of interest, and follow-up duration.

### 2.4 Evidence synthesis and appraisal

In anticipation of significant clinical diversity, we presented the available evidence for each research question in a narrative review. Two review authors (TS and AB) used the Risk of Bias Tool for RCTs, RoB-2, to assess the risk of reporting bias in our study. Finally, we evaluated the overall risk of evidence in our summarised evidence according to GRADE recommendations.

## 3 Results

### 3.1 Literature search

**Fig. 1:**
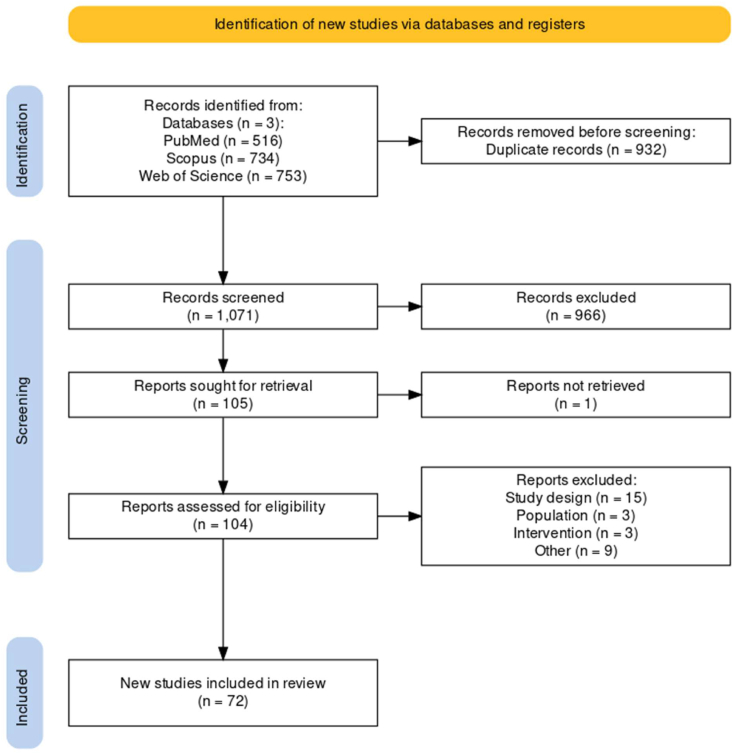
Flow chart of our study.

We conducted a literature search and found a total of 1071 unique studies. After carefully examining the title and abstract, we excluded 966 articles. Unfortunately, we were unable to retrieve the full text of one article. After reading the full text of the remaining articles, we excluded 32 additional studies, and the reasons for exclusion are described in Fig. 1. Finally, we identified 72 articles that met our criteria for the systematic review. The references to the eligible studies did not reveal any further studies.

### 3.2 Eligible studies

This systematic review is based on 72 RCTs with a total of 7701 patients (with a median of 85 patients per study). Out of these, 64 studies focused on patients with "sciatica", and the remaining eight studies were conducted on patients with "back pain and sciatica". As anticipated, there was a significant variation in the aims of the studies, measured outcomes, and tools used. The length of follow-up varied from two weeks to four years.

### 3.3 Comparison of ESI to other methods (Q1)

#### 3.3.1 ESI vs. saline injections

Eleven studies compared the efficacy of ESI over saline (Karppinen et al., 2001; Vad et al., 2002; Sayegh et al., 2009; Valat, 2003; Arden et al., 2005; Shin, 2013; Shin et al., 2015; Sinofsky, 2014; Nandi, 2017; Keorochana et al., 2018; Iversen et al., 2011)**.***Karppinen* et al., evaluated the effectiveness of ESI for sciatica and randomly assigned 160 patients to receive either a mixture of methylprednisolone and bupivacaine vs saline injection. Both treatments showed improvement during the follow-up period (Karppinen et al., 2001). At two weeks, the steroid group showed a better recovery for leg pain, straight leg raising, lumbar flexion, and patient satisfaction (Karppinen et al., 2001). However, by three and six months, the steroid group showed a rebound phenomenon and 18 patients in the steroid group and 15 in the saline group underwent surgery (Karppinen et al., 2001).

According to *Vad* et al.*'s*, research, patients who received transforaminal epidural steroid injections (TFESI) had a significantly higher success rate of 84 % after an average of 1.4 years, compared to those who received saline trigger-point injections with a success rate of only 48 % (Vad et al., 2002). The success rate was measured based on factors such as patient satisfaction, Roland-Morris score improvement, and pain reduction (Vad et al., 2002).

Similarly, *Sayegh* et al., determined the effectiveness of caudal epidural injections (CEI) containing steroid versus nonsteroid preparations in treating chronic low back pain (LBP) and sciatica (Arden et al., 2005). The patients were divided into two groups, one receiving steroid-containing CEI and the other receiving nonsteroid CEI. The results showed that CEI is effective in treating LBP and sciatica (Arden et al., 2005). Steroid-containing CEI was more effective and faster in relieving patients than nonsteroid preparations (Arden et al., 2005).

*Nandi and Chowdhery* reported similar results (Keorochana et al., 2018). The study involved two groups - one received the steroid injection (80 mg methylprednisolone), while the other received isotonic saline (Keorochana et al., 2018). Patients evaluated treatment success using a four-item satisfaction scale. Initially, the group treated with the steroid showed a significant difference in the primary outcome compared to the placebo group (Keorochana et al., 2018). However, upon completion of the study, no significant difference was observed in the primary outcome between the two groups at 12 weeks (Keorochana et al., 2018).

Improved outcomes occur irrespective of the solvent used (Shin et al., 2015). *Shin* et al.*'s study* determined the effectiveness of adding hypertonic saline instead of normal saline to the triamcinolone in the TFESI (Shin et al., 2015). The results showed that hypertonic saline provided similar short-term pain relief and improved functional outcomes within four months but had limited long-term effects (Shin et al., 2015).

*Sinofsky* et al., analyzed the relationship between concordant versus discordant pain provocation during interlaminar epidural steroid injection (ILESI) and its effects on pain reduction at follow-up (Sinofsky, 2014). The results showed that the concordant group achieved a significant decrease in self-reported pain as compared to the discordant group at 2-week follow-up (Sinofsky, 2014). There were no significant differences between the two groups in terms of improved function or reduced analgesic requirements (Sinofsky, 2014).

Another study by *Shin* et al., evaluated the effectiveness of epidural steroids in 100 patients undergoing percutaneous endoscopic lumbar discectomy (PELD) due to a herniated lumbar disc (Nandi, 2017). The results showed that epidural steroids after PELD might reduce back and leg pain while improving functional outcomes in the short-term postoperative period (Nandi, 2017). The study also revealed that epidural steroids can shorten hospital stays and the period before returning to work (Nandi, 2017). However, a similar study by Keorochana et al. failed to replicate these findings, as the ES administration with PELD did not improve postoperative pain, morphine requirements, or disability scores in the short-term and midterm periods (Iversen et al., 2011).

On the contrary, in a study by *Valat* et al., patients suffering from sciatica and LDH were randomly given three epidural injections of 2 ml prednisolone or 2 ml isotonic saline at two-day intervals (Sayegh et al., 2009). The study results showed that successful treatment was observed in 22 out of 43 patients (51 %) in the steroid group and in 15 out of 42 patients (36 %) in the control group (Sayegh et al., 2009). However, the difference between the two groups was not statistically significant. Therefore, the study concluded that epidural steroid injections do not provide any additional benefit over epidural saline injections (Sayegh et al., 2009).

Similarly, A*rben* et al. focused on 228 randomly assigned patients to receive either three lumbar ESIs of triamcinolone acetonide or interligamentous saline injections (Valat, 2003). The ESI group showed some temporary improvement over the placebo group at three weeks but no benefits at 6–52 weeks (Valat, 2003). Physical function, return to work, and surgery needs were not improved by ESIs. Repeated ESIs did not offer any additional advantages over a single injection (Valat, 2003). At the end of the study, most patients still experienced significant pain and disability, regardless of the treatment given (Valat, 2003).

Iversen et al. aimed to assess the efficacy of caudal epidural steroid or saline injection in chronic lumbar radiculopathy in the short (6 weeks), intermediate (12 weeks), and long-term (52 weeks) (Shin, 2013). The study included 138 participants who were randomized to receive subcutaneous sham injections of 2 mL 0.9 % saline, caudal epidural injections of 30 mL 0.9 % saline, and caudal epidural injections of 40 mg triamcinolone acetonide in 29 mL 0.9 % saline (Shin, 2013). The results showed that all groups improved after the interventions, but there were no statistical or clinical differences between the groups over time (Shin, 2013). Therefore, the conclusion drawn from the study was that caudal epidural steroid or saline injections are not recommended for chronic lumbar radiculopathy (Shin, 2013).

#### 3.3.2 ESI vs. “care as usual”

Five studies compared ESIs to “care as usual” (Murakibhavi and Khemka, 2011; Spijker-Huiges et al., 2014a, 2014b, 2015; Mehta et al., 2017a). The study by Murakibhavi and Khemka involved patients with chronic low back pain with sciatica (Murakibhavi and Khemka, 2011). Group A received conservative treatment such as tizanidine, diclofenac for pain, amitriptyline, bilateral skin traction, and physiotherapy, while Group B received a caudal epidural steroid injection with 20 ml normal saline, 2 ml 2 % preservative-free Xylocaine, and 2 ml (40 mg/ml) of triamcinolone acetate (Murakibhavi and Khemka, 2011). Patients were evaluated using various tools, including VAS, ODI, Beck depression inventory, and NPI questionnaires (Murakibhavi and Khemka, 2011). Pain relief was the primary index for evaluation, and the intervention group reported a higher number of patients with complete pain relief after the 6-month evaluation period (Murakibhavi and Khemka, 2011). The ODI scores significantly improved within the intervention group, and the SLRT kept improving in the intervention group from visit 2 until visit 4 (Murakibhavi and Khemka, 2011).

Spijker-Huiges et al., conducted a study to assess the effectiveness of TFESI with 80 mg of triamcinolone in 10 ml of normal saline on pain and disability, as an addition to usual care for acute lumbosacral radicular syndrome in general practice (Spijker-Huiges et al., 2014a). The study consisted of 63 patients in the acute phase of Lumbosacaral radicular syndrome and a small significant effect in favour of the intervention was found for back pain, impairment and disability score (Spijker-Huiges et al., 2014a). However, the differences were too small to be considered clinically relevant (Spijker-Huiges et al., 2014a). Therefore, the authors did not recommend implementing ESIs as an additional regular treatment option in general practice (Spijker-Huiges et al., 2014a). Additionally, the cost-utility analysis showed that the intervention group saved more societal costs due to increased productivity (Spijker-Huiges et al., 2014b, 2015).

The goal of the study by Mehta et al. was to compare conservative management and lumbar TFESIs (Mehta et al., 2017a). The study included 120 patients, who were randomly assigned to two groups: Group C (n = 60) received conservative management, which included bed rest, analgesics, and physiotherapy, while Group T (n = 60) underwent lumbar TFESIs using methylprednisolone 40 mg with 2 ml bupivacaine (Mehta et al., 2017a). Thirty minutes after the injection, two weeks, and one month, the VAS scores were found to be lower and significantly improved in Group T (Mehta et al., 2017a). Patients who received TFESI had a 98 % recovery rate of the straight leg raise test. Patients in Group T reported significantly higher satisfaction scores, and there was a reduction in drug dose intake before and after the treatment (Mehta et al., 2017a).

#### 3.3.3 ESIs vs. NSAIDs

Two studies compared ESIs to NSAIDs (Cohen, 2012; Cervera-Irimia and Tomé-Bermejo). *Cohen* et al., compared the efficacy of two epidural injections of steroids, etanercept, or saline (mixed with bupivacaine) in subacute sciatica (Cohen, 2012). The study showed that etanercept achieved a significantly smaller reduction in back pain and functional capacity than the other two groups (Cohen, 2012). On the other hand, more participants treated with epidural steroids reported 50 % or more leg pain relief and a positive global perceived effect at one month than those who received saline or etanercept (Cohen, 2012). It is uncertain, however, whether etanercept was given at a therapeutic dose (Cohen, 2012).

Cervera-Irimia et al., conducted a prospective, randomized, case-control study of a group of 46 patients with chronic low back disk pain (Cervera-Irimia and Tomé-Bermejo). Patients were randomly allocated into two groups to either receive fluoroscopy-guided CESI (CESI-group), or oral non-steroidal anti-inflammatory drugs (NSAID-group) (Cervera-Irimia and Tomé-Bermejo). The results indicate that lumbar pain, measured by the visual analog scale (VAS) and the Oswestry Low Back Pain Disability Questionnaire (ODQ), did not improve significantly during follow-up in any of the two study groups (Cervera-Irimia and Tomé-Bermejo). However, younger patients, women, patients with shorter duration of symptoms, low physical job demand, without leg pain, and sport-active, included in CESI-group showed a trend toward better results, but none reached statistical significance (Cervera-Irimia and Tomé-Bermejo).

#### 3.3.4 ESIs vs. education and spinal manipulation therapy

One study focused on the comparison of ESIs to patient education and manipulation therapy (Bronfort et al., 2004). In a pilot study conducted by Bronfort et al., 32 individuals were randomly assigned to receive one of three treatments: spinal manipulation, epidural steroid injections, or self-care education (Bronfort et al., 2004). At the end of the treatment phase, the most significant improvement was observed in the Oswestry disability score, followed by the leg pain severity, and the level of ‘bothersomeness’ of the symptoms in all three groups (Bronfort et al., 2004). After 52 weeks, the study showed that the most improvement was the leg pain severity, followed by the Oswestry disability score and the level of bothersomeness (Bronfort et al., 2004). The majority of the patients reported high levels of satisfaction due to the significant improvement in their symptoms (Bronfort et al., 2004). However, the authors failed to provide any comparisons between the three groups due to the smallnumber of enrolled patients, providing a major limitation to this study that with small number of patients being divided in the three arms of the study (Bronfort et al., 2004).

#### 3.3.5 Steroids local anaesthesia and selective nerve blocks

To investigate long-term effectiveness and ability to reduce the need for surgery, Wilson-MacDonald et al., conducted a study on 93 patients who had been offered lumbar decompression (Wilson-MacDonald et al., 2005). Patients were randomly allocated to receive either a caudal epidural injection with a local anaesthetic and steroid (40 mg bupivacaine with 80 mg of methylprednisolone) or an intramuscular injection of the same mixture and were followed up for at least two years using the Oxford pain chart and the Oswestry disability index (Wilson-MacDonald et al., 2005). Results showed that while the injection may provide short-term pain relief, it does not significantly alter long-term outcomes or reduce the need for surgery (Wilson-MacDonald et al., 2005).

A prospective randomized single-blind study by Singh et al. included eighty patients with confirmed single-level LDH divided into two groups (Singh et al., 2017). The caudal group received three injections of steroid mixed with local anaesthetic, while the selective nerve root block group received a single injection of steroid mixed with a local anaesthetic agent (Singh et al., 2017). The study found that the caudal epidural block was an easy and safe method that provided better pain relief and improvement in functional disability than a selective nerve root block (Singh et al., 2017). The reduction in pain and improvement in the Oswestry Disability Index (ODI) were more significant and long-lasting in the caudal group (Singh et al., 2017). Specifically, the reduction in pain was more than 50 % in the selective nerve root block group up until 6 months, while in the caudal group, more than 50 % reduction of pain was maintained until 1 year (Singh et al., 2017).

#### 3.3.6 ESIs vs. ozone mixture gas

Three strides compared ESI to ozone therapy (Bonetti et al., 2005; Zambello et al., 2006; Krahulik et al., 2023). *Bonetti* et al., compared the clinical outcomes of 306 patients afflicted with sciatic nerve pain who received either intraforaminal O2O3 mixture gas or epidural steroid infiltration at short-, medium-, and long-term follow-up (Bonetti et al., 2005). After one week, most patients experienced complete pain relief, irrespective of the treatment modality they received (Bonetti et al., 2005). However, at the six-month follow-up, the O2-O3 treatment was found to be significantly more effective (Bonetti et al., 2005). Similarly, *Zambello* et al., conducted a study to compare the effectiveness of epidural injection of steroids and intramuscular paravertebral infiltration of an O2O3 mixture in treating symptomatic herniated discs in the lumbar spine (Zambello et al., 2006). The short-term results showed that pain remission was achieved in 59 % of patients treated with steroids and 88.2 % of patients treated with O2O3 (Zambello et al., 2006). Equally important, in the long term, O2O3 had better outcomes than epidural steroid injections, with 77.1 % versus 47.3 % of patients experiencing excellent or good results (Zambello et al., 2006). A study by Krahulik et al. evaluated the effectiveness of different treatments for radicular pain in 150 patients who did not respond to conservative management (Krahulik et al., 2023). Betamethasone, methylprednisone, and ozone were used as treatment agents and all three showed a statistically significant effect (Krahulik et al., 2023). However, betamethasone was found to be more effective than the other two in reducing leg pain after therapy (Krahulik et al., 2023).

#### 3.3.7 ESIs vs. other minimally invasive interventions

ESIs were compared to several minimally invasive autologous conditioned serum, epiduroscopy, PRF, and plasma disc decompression. A clinical trial by Hashemi et al. compared the efficacy of autologous conditioned serum (ACS) and epidural corticosteroid injection for treating lumbar radicular pain associated with disc herniation (Hashemi et al., 2022). The results showed that both treatments were effective in reducing pain intensity, but ACS was superior to triamcinolone in the final evaluation at six months (Hashemi et al., 2022). No specific complications were reported with the use of ACS (Hashemi et al., 2022). The study provides valuable insights into the efficacy and safety of ACS therapy for managing lumbar radicular pain (Hashemi et al., 2022).

Dashfield et al. focused on 60 patients with a history of sciatica to investigate the impact of steroid placement within the epidural space (Dashfield et al., 2005). Both targeted epidural local anaesthetic and local anaesthetic and steroid treatment during epiduroscopy were found to be effective in reducing pain intensity, anxiety, and depression. However, the study failed to compare the two techniques directly (Dashfield et al., 2005).

Lee et al. compared the effectiveness of pulsed radiofrequency (PRF) and TFESI for treating radicular pain due to disc herniation (Lee et al., 2016). Forty-four eligible patients were enrolled, and 38 subjects were randomly assigned to receive either PRF (PRF group; n = 19) or additional TFESI (TFESI group; n = 19) and were then followed for 2, 4, 8, and 12 weeks (Lee et al., 2016). Pain intensity was evaluated by VAS, and functional disability was assessed by ODI and NDI. The study found that PRF administered to a dorsal root ganglion might be as effective as TFESI in terms of attenuating radicular pain caused by disc herniation, and its use would avoid the adverse effects of steroids (Lee et al., 2016).

A single clinical study by Gerszten et al. compared the effectiveness of plasma disc decompression (PDD) and fluoroscopy-guided Transforaminal Epidural Steroid Injection (TFESI) for patients with radicular pain (Gerszten et al., 2010). Ninety patients were enrolled and randomly assigned to receive either PDD (46 patients) or TFESI (44 patients) (Gerszten et al., 2010). The study observed the clinical outcomes of PDD and TFESI over the course of two years (Gerszten et al., 2010). The results showed that patients in the PDD group had a significantly greater reduction in leg pain scores and improved Oswestry Disability Index and 36-Item Short Form Health Survey (SF-36) scores than those in the TFESI group (Gerszten et al., 2010). Additionally, more patients in the PDD group remained free from having a secondary procedure following the study procedure (Gerszten et al., 2010).

#### 3.3.8 ESIs vs. surgery

The NErve Root Block VErsus Surgery (NERVES) trial by Wilby et al. compared microdiscectomy's clinical effectiveness and cost-effectiveness with transforaminal epidural steroid injection to manage radicular pain (Wilby et al., 2021). The study recruited 163 participants from 11 UK NHS outpatient clinics (Wilby et al., 2021). The study found no difference between the two treatments for the Oswestry Disability Questionnaire scores, visual analog scores for leg and back pain, modified Roland–Morris score and Core Outcome Measures Index score up to 54 weeks (Wilby et al., 2021). However, microdiscectomy had an incremental cost-effectiveness ratio of £38,737 per quality-adjusted life-year gained and a probability of 0.17 being cost-effective at a willingness to pay a threshold of £20,000 per quality-adjusted life-year (Wilby et al., 2021).

A study by Buttermann et al. evaluated the effectiveness of epidural steroid injection in treating patients with a large, symptomatic lumbar herniated nucleus pulposus who were surgical candidates (Buttermann). Out of 169 patients with a large herniation, 100 were randomly assigned to receive either an epidural steroid injection or discectomy (Buttermann). The study found that discectomy was superior to ESIs as it led to a rapid decrease in symptoms in up to 98 % of the patients which remained over the various follow-up periods (Buttermann). On the other hand, only 42 %–56 % of the 50 patients who had undergone the epidural steroid injection reported that the treatment had been effective (Buttermann). Nevertheless, patients who did not respond to epidural steroid injection but underwent discectomy later did not show any adverse effects from the delay in surgery caused by the trial (Buttermann).

### 3.4 Comparison between various ESI approaches (Q2)

**Eight studies compared the safety and efficacy of the TFESI to the ILESI approaches**. Initially, Thomas et al. compared the effectiveness of transforaminal and interspinous epidural corticosteroid injections in treating radiculopathy involving 31 patients who received either fluoroscopy-guided transforaminal or blindly performed interspinous injections (Thomas et al., 2003). The outcomes indicated that the transforaminal group showed significant improvement compared to the interspinous group in various measures, including pain relief, daily activities, work, and leisure activities (Thomas et al., 2003). Moreover, Rados et al. reported that patients who received epidural steroid injections via the transforaminal approach showed lower levels of depression and anxiety due to a greater reduction in pain compared to the interlaminar group (Radoš et al., 2018). Although there was no significant difference between the two groups, the sleep quality was found to be higher in the transforaminal group (Radoš et al., 2018). The superior results achieved by TFESI were attributed to the more ventral epidural steroid placement and were replicated by several studies Aref et al. (2007), (Gharibo, 2011; Maadawy et al., 2018; Kumar et al., 2023). Nevertheless, Candido et al. (2008) compared the parasagittal interlaminar (PIL) and transforaminal (TF) approaches in placing contrast into the anterior epidural space (Candido et al., 2008). The study included sixty adult patients with low back pain and unilateral radiculopathy from LDH, with 30 patients in each group (Candido et al., 2008). The results showed that the PIL approach is superior to the TF approach in terms of anterior epidural spread, reduction in fluoroscopy times, and improved spread grade (Candido et al., 2008). To make things more complicated, an RCT conducted by Ghai et al. reported that the ventral epidural spread was found to be comparable in both groups (Ghai, 2014). While no major complications were encountered in either group, initial intravascular spread of contrast was observed in three patients in the TF group (Ghai, 2014). The study concluded that both approaches resulted in effective pain relief and improvement in back-related functionality, with no significant difference between the two groups (Ghai, 2014).

**Four studies compared the efficacy of caudal epidural steroid injections to the transforaminal and interlaminar approaches** (Ackerman and Ahmad, 2007; Kamble et al., 2016; Ozturk et al., 2023; Akram et al., 2021). Ackerman et al. conducted a study to evaluate the effectiveness of caudal (C), interlaminar (IL), and transforaminal (TF) epidural steroid injections in treating radicular pain associated with LDH in 90 adult patients (Ackerman and Ahmad, 2007). The results showed that the TF route was significantly more effective than the C and IL routes in providing pain relief (Ackerman and Ahmad, 2007). Once again, the study attributed their results to the higher chances of steroid placement in the ventral epidural space when the TF method is used (Ackerman and Ahmad, 2007). The study by Kamble et al. encompassed a sample of 90 patients who were randomly assigned to one of three groups, each with 30 patients (Kamble et al., 2016). The results indicated that the transforaminal steroid injection group demonstrated a significantly greater improvement in pain and ODI scores when compared with the other two groups (Kamble et al., 2016). On the other hand, there was no significant difference in the change of pain and ODI scores between the interlaminar and caudal routes at any time of assessment (Kamble et al., 2016). Likewise, Akram et al. compared the outcomes of caudal and interlaminar epidural steroid injections in treating sciatica patients (Akram et al., 2021). The study showed that interlaminar epidural steroid injections were more effective than caudal ESIs (Akram et al., 2021). Conversely, Ozturk et al. compared the effectiveness of caudal and transforaminal epidural injections in patients with unilateral S1 radiculopathy (Ozturk et al., 2023). The study included 60 patients, with both groups showing significant improvement in pain and disability scores at 3 weeks and 3 months compared to baseline, with no significant difference between the groups (Ozturk et al., 2023). Both approaches were effective in managing S1 radiculopathy, with the CESI approach requiring less fluoroscopy time and radiation exposure, and hence, being a safer and potentially more cost-effective option (Keorochana et al., 2018).

**Three additional studies focused on minor approach variations** (Jeong et al., 2007)**.** Jeong et al. conducted a study to determine the efficacy of transforaminal epidural steroid injection (TFESI) in treating lumbosacral radiculopathy, with a specific emphasis on the injection site (Jeong et al., 2007). The findings showed that the preganglionic group had a significantly better treatment response (88.4 %) compared to the ganglionic group (70.9 %) in the short term (Jeong et al., 2007). Additionally, the injection site was the only significant predictor of outcome at short-term follow-up (Jeong et al., 2007). Makkar et al. compared epidural steroid injections (ESIs) given through three different routes (midline interlaminar, parasagittal interlaminar, and transforaminal) in patients with unilateral lumbar radiculopathy (Makkar, 2019). Sixty-five patients received one of the three types of injections (Makkar, 2019). The results showed that the PIL and TF groups had significantly lower VAS scores compared to the MIL group. The PIL approach was found to be equivalent to TF and superior to MIL in terms of effective pain relief and decrease in disability (Makkar, 2019). The study suggested that the PIL approach is a technically superior route with fewer complications and greater drug delivery into the ventral epidural space compared to the TF route (Makkar, 2019). Likewise, Kumar et al. compared the efficacy of midline and parasagittal approaches for interlaminar ESI in treating symptomatic lumbar intervertebral disc herniation (Kumar et al., 2022). Both techniques were effective in providing good analgesia, with no significant difference between the two groups (Kumar et al., 2022).

**Finally, two studies focused on the appropriate target of the TFESI** (Ali et al., 2021; Singh et al., 2022)**.**Ali et al. provide insights into the efficacy of transforaminal epidural steroid injection (TFESI) in patients with lumbar radiculopathy using conventional versus Kambin's triangle approaches (Ali et al., 2021). After the procedure, both groups exhibited a significant improvement in the pain score and patient satisfaction, as observed at four and eight weeks post-procedure (Ali et al., 2021). However, no statistically significant difference in pain score or patient satisfaction was observed between the two groups (Ali et al., 2021). Similarly, Singh et al. conducted a randomized, double-blind comparative study on 40 patients suffering from lumbar radicular pain aiming to compare the efficacy of the conventional transforaminal and Kambin's triangle approach (Singh et al., 2022). Both approaches of transforaminal epidural steroid injection were effective in significantly reducing pain and increasing functional status (Singh et al., 2022).

### 3.5 Compare ESI to analgesia (Q3)

Manchikanti et al. conducted a study to evaluate the efficacy of fluoroscopically directed caudal ESIs over epidural analgesia for the management of patients with disc herniation and radiculitis (Manchikanti et al., 2011). They randomly assigned 120 patients to receive either caudal epidural injections with local anaesthetic or with local anaesthetic mixed with steroids (Manchikanti et al., 2011). The study found that both treatments were effective, with the steroid group showing better relief with the first and second procedures (Manchikanti et al., 2011). Overall, the study suggests that caudal epidural injections may be effective in managing pain in these patients (Manchikanti et al., 2011).

A study by Ghai et al. compared the effectiveness of epidural injections of local anaesthetic alone (lidocaine) and a mixture of local anaesthetic with steroids (lidocaine plus methylprednisolone) using an interlaminar approach (Ghai, 2015). The one-year follow-up study involved 69 patients and showed that adding a steroid to local anaesthetics for epidural injections may provide better pain relief for managing chronic low back pain with unilateral lower radicular pain, although local anaesthetic alone was also effective (Ghai, 2015).

A study by Ng et al. aimed to determine the effectiveness of corticosteroids in periradicular infiltration for chronic radicular pain (Ng et al., 2005). Eighty-six patients were randomly selected to receive a single injection with bupivacaine and methylprednisolone or bupivacaine only (Ng et al., 2005). After three months, there was no significant difference between the groups in terms of changes in pain levels or disability (Ng et al., 2005). Both groups showed clinical improvement, but corticosteroids did not provide additional benefit (Ng et al., 2005).

The study by Tafazal et al. assessed the effectiveness of ESI compared to analgesia in treating patients with radicular pain due to lumbar disc herniation or lumbar spinal stenosis (Tafazal et al., 2009). The authors found that while there were no significant differences in standard outcome measures, such as pain intensity and back-related function between patients who received epidural injections of bupivacaine alone and those who received bupivacaine and methylprednisolone at the 3-month follow-up, corticosteroids could prevent the need for subsequent interventions (Tafazal et al., 2009). The study evaluated the requirement for additional root blocks or surgery at a minimum of 1-year post-injection (Tafazal et al., 2009).

A randomized controlled trial by Okmen et al. was conducted on 98 patients, comparing the efficacy of interlaminar epidural steroid administration with 10 mL 0.25 % bupivacaine to a group that also received 40 mg methylprednisolone (Ökmen and Ökmen, 2017). The study found that patients who received steroids had lower VAS and ODI scores, indicating a higher success rate of the procedure (Ökmen and Ökmen, 2017). Age and BMI did not affect the success of the treatment. However, the authors suggested that further research is needed to determine the optimal dose of steroids and the dispersion of ILESI in the epidural area (Ökmen and Ökmen, 2017).

Ter Meulen et al. assessed the efficacy of TFESIs in 141 individuals experiencing acute sciatica (Ter Meulen et al., 2023). The participants were arbitrarily allocated to three groups: TFESI with a combination of methylprednisolone and levobupivacaine (group 1), usual care and TFESI with levobupivacaine solution (group 2), and oral pain medication with or without physiotherapy (group 3) (Ter Meulen et al., 2023). The results showed no significant differences among the groups, except for a reduction in leg pain in intervention group 1 (Ter Meulen et al., 2023). Notably, both intervention groups had lower opioid usage (Ter Meulen et al., 2023).

### 3.6 Optimal ESI protocol (Q4)

Five studies aimed to determine the most suitable steroid for ESIs (Cocelli et al., 2009; Khan et al., 2010; Park et al., 2010; Datta and Upadhyay, 2011; Kennedy et al., 2014). Cocelli et al. conducted a study on 70 patients with unilateral radiculopathy, comparing the efficacy of epidural betamethasone and triamcinolone injections (Cocelli et al., 2009). Both steroids resulted in significant improvement in the first week of treatment (Cocelli et al., 2009). However, triamcinolone achieved significantly lower VAS values at the first, second, and sixth weeks (Cocelli et al., 2009). Thus, triamcinolone was found to be preferable over betamethasone for an ESI procedure due to its superior short-term efficacy (Cocelli et al., 2009). Another study conducted by Khan et al. aimed to assess the effectiveness of epidural steroid injections using methylprednisolone and triamcinolone via the lumbar and caudal route for patients with sciatica (Khan et al., 2010). The results showed that 78.43 % in the caudal group and 82.6 % in the lumbar group had excellent to good outcomes (Khan et al., 2010). A significant difference was found in the improvement of low backache by both routes and both drugs (Khan et al., 2010). However, the difference was statistically insignificant among the two steroid groups and two routes (Khan et al., 2010). In addition, Park et al. conducted a study to compare the effectiveness of nonparticulate and particulate steroids in treating LDH (Park et al., 2010). One hundred and six patients were given either dexamethasone or triamcinolone acetate lumbar transforaminal ESIs (Park et al., 2010). Triamcinolone was found to be more effective in reducing low back pain associated with sciatica than dexamethasone (Park et al., 2010). Likewise, a study by Kennedy et al. enrolled 78 subjects and found that both triamcinolone and dexamethasone resulted in significant improvements in pain and function at two weeks, three months, and six months, without any significant differences between the two groups (Kennedy et al., 2014). The study revealed that corticosteroid injections are an effective treatment for acute radicular pain that often requires only one or two injections for symptomatic relief (Kennedy et al., 2014). Datta and Upadhyay conducted a study to compare the efficacy of caudal methylprednisolone acetate with triamcinolone acetonide and dexamethasone acetate for pain relief in sciatica associated with LDH (Datta and Upadhyay, 2011). The study included 163 patients divided into four groups, including a control group (Datta and Upadhyay, 2011). Pain relief was present in all groups within three weeks, with no significant difference between the groups (Datta and Upadhyay, 2011). By the six and 12 weeks, the three steroid groups had significant pain relief, with both methylprednisolone and triamcinolone groups showing greater improvement in the finger-to-floor distance (Datta and Upadhyay, 2011). Overall, pain relief was significantly better in the steroid group than in the control group at all follow-up evaluations (Datta and Upadhyay, 2011). The study suggested that ESI therapy is an effective and safe treatment option for sciatica associated with LDH (Datta and Upadhyay, 2011).

One RCT identified the optimal dosage of ESIs (Owlia et al., 2007), other two the targeted concentration (Makkar, 2018; Aref et al., 2011), and a fourth study the ideal delivery rate (Thiengwittayaporn et al., 2021). A study by Owlia et al. compared the effects of administering ESI with 80 mg versus 40 mg of methylprednisolone for lumbar radicular pain involving 84 patients (Owlia et al., 2007). Results showed that 75 % of patients in both groups showed remarkable improvement in pain after one month, concluding that ESI with a low dose (40 mg) of methylprednisolone is as effective as a high dose (80 mg) with comparable results but a higher safety profile (Owlia et al., 2007). On the other hand, Makkar et al. conducted a double-blind trial on 60 patients to investigate the impact of increasing epidural drug volume on pain relief in lumbar ESI (Makkar, 2018). Patients were divided into three groups (4 mL, 6 mL, and 8 mL) (Makkar, 2018). Results showed no significant difference in effective pain relief between the three groups after six months (Makkar, 2018). The study concluded that an increase in the volume of the injectate from 4 mL to 8 mL did not increase the efficacy of interlaminar ESI (Makkar, 2018). A study by Aref et al. compared the efficacy of different volumes/concentrations of ESIs in treating 60 ASA patients with unilateral radiculopathy (Aref et al., 2011). Patients who received low volumes and high concentrations of corticosteroid through the transforaminal epidural approach showed significantly better short-term pain improvement and a lower incidence of the need for surgical intervention when compared to those treated with a diluted corticosteroid solution (Aref et al., 2011). Moreover, Thiengwittayaporn et al. conducted a study comparing the clinical outcomes and complications of caudal epidural steroid injections (CESI) at two different rates (40 mL/min and 20 mL/min) in patients with lumbosacral radiculopathy (Thiengwittayaporn et al., 2021). The study involved ninety patients, and the results showed no significant difference in outcomes between the two groups, except for the pain at the injection site (Thiengwittayaporn et al., 2021). Patients who received fast injections experienced more pain than those who received slow injections, suggesting that the slow injection rate should be considered (Thiengwittayaporn et al., 2021).

Five studies were conducted to investigate methods for improving ESI outcomes. These methods included co-administering amitriptyline or adding clonidine to the mixture, using pulsed radiofrequency, administering steroids via a targeted catheter, and performing the injection under sedation. A study by Pirbudak, et al., n.d. compared the effectiveness of ESI alone versus ESI combined with amitriptyline in treating back and leg pain caused by LDH (Pirbudak, et al., n.d.). Both treatments were found to be effective in both groups after six months, but those who received EPSI combined with amitriptyline reported a better quality of life (Pirbudak, et al., n.d.). Likewise, Tauheed et al. compared the effectiveness of low doses of clonidine combined with steroids versus methylprednisolone alone for transforaminal injection in lumbosacral radiculopathy (Tauheed et al., 2014). The study showed that adding 1mcg/kg clonidine to 60 mg methylprednisolone in transforaminal epidural injections provided better pain relief with practically no significant side effects (Tauheed et al., 2014). However, the lack of a placebo group limits the study (Tauheed et al., 2014). In a clinical trial by Napoli et al. combined pulsed radiofrequency (PRF) and transforaminal epidural steroid injection (TFESI) treatment was found to be significantly more effective in relieving pain and improving disability than steroid injection alone (Napoli et al., 2023). The study had 351 participants, reported a low rate of adverse events, and provided important implications for managing sciatica caused by lumbar disk herniation (Napoli et al., 2023). A study by Sencan et al. compared the effectiveness of administering transforaminal epidural steroid injections (TFESI) with or without sedation (Sencan et al., 2019). Results showed that patient and physician satisfaction was significantly higher in the sedation group, and periprocedural pain levels were significantly lower (Sencan et al., 2019). The researchers concluded that coadministration of TFESI with sedation improves patient and physician satisfaction (Sencan et al., 2019). Yin et al. conducted a study on the effectiveness and safety of targeted indwelling catheters for administering ESI combined with manipulative therapy in patients with LDH radiculopathy (Yin et al., 2018). The study included 85 eligible patients who were randomly divided into the Catheter Group (N 43) and the No-Catheter Group (N 42) (Yin et al., 2018). Both methods were effective in reducing pain intensity and functional disability compared to pre-treatment, with the Catheter Group showing more significant results in short-term follow-up (Yin et al., 2018).

### 3.7 Comparison of guiding techniques (Q5)

Three RCTs studied the role of ultrasound (US) in guiding ESI in LDH, whereas no RCT focused on CT guidance (Darrieutort-Laffite et al., 2015; Elashmawy et al., 2020; Park et al., 2013). A study conducted by Darrieutort-Laffite aimed to determine if using ultrasound (US) to select the best puncture level could make epidural steroid injections easier under challenging cases (Darrieutort-Laffite et al., 2015). The study included 80 patients who were randomized into two groups, with and without US (Darrieutort-Laffite et al., 2015). The results revealed a positive correlation between the depth of the epidural space and body mass index (BMI), and a negative correlation between age and interspinous space size (Darrieutort-Laffite et al., 2015). Interestingly, the study reported that using US guidance did not significantly reduce pain intensity during the procedure (Darrieutort-Laffite et al., 2015). The authors postulated that the use of US-guided ESIs was useful, even in obese and elderly patients and allowed for adequate visualization of the epidural space (Darrieutort-Laffite et al., 2015). The study by Elashmawy et al. compared the effectiveness of ultrasound (US) -guided and fluoroscopy (FL)-guided caudal epidural steroid injection (CESI) in treating refractory lumbar disc prolapse with radiculopathy (Elashmawy et al., 2020). Both groups of 68 patients showed significant improvement in various tests at 1-month and 3-month post-injection evaluation compared to baseline recordings (Elashmawy et al., 2020). The study found that US is an excellent guide for CESI, producing similar treatment outcomes to FL-guided CESI (Elashmawy et al., 2020). Another study by Park et al. compared US-guided caudal epidural steroid injections with FL-guided epidural steroid injections for unilateral radicular pain in the lower lumbar spine (Park et al., 2013). A total of 120 patients were randomly assigned to either group. The results showed similar improvements in short-term pain relief, function, and patient satisfaction with both ultrasound and fluoroscopic guidance (Park et al., 2013). However, the study indicated that the US approach with colour Doppler mode helped to avoid intravascular injection-induced complications (Park et al., 2013).

### 3.8 ESIs as a predictive factor for sciatica outcome and progression to surgery (Q6)

A single study was conducted to investigate the role of ESIs in the progression to surgery for patients with LDH (Radcliff et al., 2012). The study was part of the Spine Patient Outcomes Research Trial (SPORT), a multicentre study on the effectiveness of operative treatment for LDH (Radcliff et al., 2012). It compared 154 patients who received ESIs to 453 patients who did not receive ESIs (Radcliff et al., 2012). The study found that patients who were treated with ESI did not show any improvement in short or long-term outcomes compared to those who did not receive ESI treatment. However, a higher number of patients in the ESI group chose nonsurgical treatment, but this was probably influenced by their initial desire to avoid surgery (Radcliff et al., 2012). The available evidence originates from a study with a low risk of bias.

## 4 Discussion

### 4.1 Summary of main findings

Our review of 72 studies found that epidural steroid injections can significantly relieve pain and improve function in sciatica, outperforming other treatments. However, ESIs are less effective than pulsed radiofrequency and ozone therapy in the short term, and their effects do not persist long-term. Surgical intervention remains superior. The transforaminal approach appears most effective, and adjunct steroids, low-dose corticosteroids, and dilute solutions can enhance ESI efficacy and safety. US-guidance is useful in challenging cases, enabling epidural visualization, though it did not reduce procedural pain. US-guided ESI and CESI are viable options, avoiding intravascular complications.

### 4.2 Comparison with the literature

The effectiveness of ESI over other non-surgical treatments from our review is in agreement with relevant systematic reviews and meta-analyses (Manchikanti et al., 2021; Verheijen et al., 2021; Shelton et al., 2022; Yang et al., 2020; Bhatia et al., 2016). Notably, Oliveira et al. conducted a meta-analysis of 25 trials with 2470 participants, finding that ESIs were more effective than placebo in reducing short-term leg pain and disability, and may slightly reduce overall short-term pain (Oliveira et al., 2020). However, the quality of evidence was moderate, possibly due to trial design and inconsistency (Oliveira et al., 2020).Liu et al. compared the efficacy of discectomy, non-surgical treatments, and ESIs for sciatica (Liu et al., 2023). Once again, the evidence suggested that discectomy is superior to non-surgical treatment or epidural steroid injections in reducing leg pain and disability (Liu et al., 2023). However, the benefits of discectomy may diminish over time, and the risk of adverse events was similar between discectomy and non-surgical treatment (Liu et al., 2023). Although the review focused more on epidural steroid injections, the findings are consistent with the study by Liu et al. (2023).

The existing literature suggests a growing interest in comparing the efficacy of different epidural steroid injection techniques, such as transforaminal, interlaminar, and caudal approaches. A review by Chang-Chien et al. analyzed eight studies, including five prospective and three retrospective, with a total of 506 patients (Chang-Chien et al., 2014). The analysis of the prospective studies showed that TFESI was slightly more effective than ILESI in reducing pain at the 2-week follow-up, but there was no difference at one or six months. Both treatments were effective in reducing pain and improving functional scores in unilateral lower spine-related pain (Chang-Chien et al., 2014). Another review by Lee et al. compared the effectiveness of TFESI and CESI, including six studies (Lee et al., 2018). Four studies supported the superiority of TFESI, one showed no significant difference, and one supported the superiority of CESI to TFESI (Lee et al., 2018). However, a meta-analysis indicated short-term and long-term trends toward better clinical efficacy with TFESI compared to CESI, although the evidence level was low due to inconsistency and imprecision (Lee et al., 2018). Overall, the available evidence suggests a marginally superior efficacy of TFESI over the other two approaches, likely due to the optimized steroid infiltration in the ventral epidural compartment.

The existing literature highlights the importance of optimizing the ESI protocol. According to Mehta et al. nonparticulate steroids are the recommended choice for patients experiencing radicular pain during lumbar TFESI (Mehta et al., 2017b). However, there is insufficient evidence to suggest a specific steroid preparation for lumbar ILESI (Mehta et al., 2017b). Given the risk of severe complications associated with particulate steroids, the authors recommend using nonparticulate steroids as the primary approach when performing ESIs (Mehta et al., 2017b).

The evidence suggests that fluoroscopy-guided and ultrasound-guided epidural steroid injections can be effective in managing back pain, though their relative superiority remains unclear. Studies have found no significant differences in pain relief and complications between FL-guided and US-guided ESIs, but FL-guided injections may be more effective for improving functionality (Viderman et al., 2023). Additionally, US-guided lumbar facet joint injections have demonstrated feasibility, accuracy, and clinical efficiency comparable to low-dose CT-guided injections (Ye et al., 2018). However, the role of CT guidance in ESI for sciatica is understudied, and both US and FL-guided ESIs are most useful in challenging cases (Ye et al., 2018).

The role of epidural steroid injections in predicting the need for surgery is not well-studied. A review by Nagington et al. found limited evidence, with studies of low quality and inconsistent methods, on prognostic factors for outcomes after ESI (Nagington et al., 2023). The most commonly assessed factors included pain, function, imaging, demographics, health, and injection characteristics, but no factors were consistently associated with outcomes (Nagington et al., 2023). This highlights a significant gap in the evidence and the need for further high-quality prospective research to guide clinical practice and improve patient care (Nagington et al., 2023). Our review suggests that identifying responders to ESI may help identify patients less likely to require surgery.

### 4.3 The importance of ESI in ManagingSciatica

The existence of 72 randomized controlled trials involving 7701 patients underscores the significant scientific and clinical interest in the role of epidural steroid injections in managing sciatica. These studies have been consistently conducted without a decreasing pace from as early as 2001 to 2023. Notably, there are large variations in the selected populations and outcome measures used to evaluate ESIs. While initial studies established the foundational role of ESIs in sciatica management, the sheer volume of research also suggests that the results are not entirely satisfactory and require further exploration. Consequently, numerous recent studies have attempted to optimize the approach, guidance method, preferred steroid, and dosage in order to maximize the benefits of ESIs for patients with sciatica. It becomes apparent that studying ESIs is more important than ever.

### 4.4 Gaps in the literature

The existing literature has provided valuable insights, yet several critical areas warrant further investigation. Firstly, the criteria for selecting patients who respond favorably to epidural steroid injections remain to be elucidated. Determining the patient subpopulation most likely to benefit from ESIs is pivotal for personalized treatment planning and reducing unnecessary interventions. Secondly, the optimal ESI protocol, including the choice of steroid, volume, and frequency of injections, has yet to be conclusively established. Thirdly, the long-term implications of ESIs on the natural history of sciatica and their potential interactions with other interventions, such as physical therapy and surgery, are not well understood. Fourthly, the criteria for assessing the therapeutic success of ESIs are poorly defined. The wide array of outcome measures used across studies, encompassing pain, function, quality of life, and patient satisfaction, impedes the ability to compare and synthesize the evidence. Finally, the reporting of adverse events associated with ESIs is inconsistent. The literature lacks comprehensive documentation and analysis of the incidence and severity of adverse events linked to different ESI techniques and formulations. Addressing these critical gaps through well-designed, methodologically rigorous studies will be essential to optimize the clinical utility of epidural steroid injections in managing sciatica.

### 4.5 Key research barriers

Several barriers are hindering the research showing the role of ESIs in managing sciatica. It is important to acknowledge these barriers and consider them when designing future studies and clinical practice guidelines. The heterogeneous nature of the patient population, with varying underlying etiologies, severity, and radiographic findings, poses a significant challenge in establishing consistent inclusion criteria and outcome measures. Furthermore, the invasive nature of ESIs and the inherent risks associated with the procedure, such as infection, bleeding, and neurological complications, raise ethical concerns and necessitate cautious consideration of patient selection and safety monitoring. The placebo effect, which is known to be substantial in ESI trials, can introduce significant bias and confound the interpretation of treatment effectiveness. Additionally, the lack of standardized protocols for ESI administration, including the choice of steroid, volume, and frequency of injections, limits the ability to compare and synthesize the existing evidence.

## 5 Limitations

It is worth noting that our study has some limitations that should be taken into account while interpreting the findings. We restricted our search to Randomized Control Trials (RCTs) to ensure as high a quality of data included, but this approach resulted in a smaller pool of eligible studies for some of our inquiries; nonetheless, a respectable amount of data was available, allowing evidence synthesis from 72 RCTs and a total of 7701 patients.We only included studies published in English. A singular literature search was conducted for multiple queries, and the studies identified exhibited a significant degree of heterogeneity in outcome reporting. Lastly, it is important to mention that we conducted a qualitative review of our findings and did not carry out quantitative analysis to summarize the results.

## 6 Conclusions

Our systematic review discusses the efficacy of ESIs in treating sciatica. It highlights that ESIs can significantly alleviate pain and improve functional outcomes for patients with sciatica, surpassing other available treatments. However, surgery is considered a superior option compared to ESIs. We also found that TFESI seems to have superior outcomes when compared to ILESI and CESI. The article also discusses various methods for improving ESI outcomes, including co-administering amitriptyline or adding clonidine to the mixture, using pulsed radiofrequency, and administering ESIs at slow injection rates. Additionally, US-guided ESIs are particularly useful in challenging cases, enabling adequate visualization of the epidural space and avoiding intravascular complications.

## Declaration of competing interests

The authors declare no competing interests.
